# Liver and Renal Impairments in COVID-19 Patients of Madinah City of Saudi Arabia: A Cross-Sectional Study (2020)

**DOI:** 10.7759/cureus.39409

**Published:** 2023-05-23

**Authors:** Walaa Mohammedsaeed, Ibrahim Ahmedseedi, Ziab Alahmadey

**Affiliations:** 1 Department of Medical Laboratory Technology, Faculty of Applied Medical Science, Taibah University, Madinah, SAU; 2 Department of Internal Medicine, Ohud Hospital Madinah, Madinah, SAU; 3 Laboratory Department, Ohud Hospital Madinah, Madinah, SAU

**Keywords:** chronic obstructive pulmonary disorder, albumin, covid-19, aspartate aminotransferase, alanine aminotransferase

## Abstract

Purpose

Most of the research on severe acute respiratory syndrome coronavirus 2 (SARS-CoV-2) (coronavirus disease 2019 [COVID-19]) has mainly focused on the lungs as a key organ involved in the disease, while very little data is available regarding the involvement of other organs including the liver and kidneys, which are also reported to be severely affected by the disease. The objective of this study was to analyze the effect of COVID-19 disease on liver and kidney functions and to determine their association with the severity and mortality of the disease. This was a retrospective cross-sectional analysis of medical records.

Methods

A total of 100 confirmed COVID-19 adult patients from Madinah, Saudi Arabia hospitalized between April 28 and June 30, 2020, were included and categorized into asymptomatic, mild to moderate, and severely ill patients. We analyzed the clinical status of liver and renal functioning in all three groups.

Results

Most patients (51%) were diagnosed with mild to moderate disease, 27% of patients were severely ill and 22% of patients were asymptomatic. The liver and renal functional analysis showed that the severity of the COVID-19 patients was significantly associated with renal impairments exhibiting higher levels of creatinine and urea (P<0.05) with high levels of liver enzymes as indicators for liver damage.

Conclusion

We concluded from the present study that severely ill COVID-19 patients were more prone to have abnormal liver and renal functions. The present findings, however, demand further study of the association between liver and kidney impairments with COVID-19 infection for better clinical management.

## Introduction

The recent severe acute respiratory syndrome coronavirus 2 (SARS-CoV-2) (coronavirus disease 2019 [COVID-19]) that started in Wuhan, China, is a crucial international health catastrophe of the present time and one of the greatest healthcare challenges the world is facing currently. Across the six World Health Organization (WHO) regions, more than 11 million new cases and just over 43,000 new deaths were reported. As of 13 March 2022, more than 455 million confirmed cases and over 6 million deaths have been reported globally [1]. The Ministry of Health has reported 749,730 cases of COVID-19 in the Kingdom of Saudi Arabia until 13 March 2022.

The clinical indications of the disease usually are fever, cough, fatigue, and other signs of respiratory tract infections. In severely ill patients, individuals show symptoms of pneumonia with atypical computed tomography (CT) chest scan, related to complications of severe acute respiratory distress syndrome, acute cardiac injury, and renal failure leading eventually to death.

Recent research has revealed that more than half of COVID-19 patients showed erratic levels of liver and renal disorder [2]. Several COVID-19 patients with pneumonia have shown renal injury, and the autopsy results of some patients who died from COVID-19 have shown renal damage sometimes [3,4]. However, little is known about the clinical characteristics of kidney-related complications. Furthermore, a histopathology study of liver biopsy samples from a patient who died from COVID-19 revealed moderate microvascular steatosis and minor lobular and portal activity, signifying that SARS-CoV-2 could be the underlying cause of this liver damage [5]. Nevertheless, recent research has shed light on the impact of COVID-19 on various body organs, but still, there is a paucity of data related to the clinical outcomes of this disease. Hence, the aim of the present study was to analyze the clinical significance of liver and renal parameters in COVID-19 patients admitted to the referral hospital in the Madinah region of Saudi Arabia.

In the present study, we sought to analyze the clinical significance of liver and renal function changes and evaluate their relationship with the disease progression in COVID-19 patients.

This article was previously posted to the Research Square preprint server on August 2020, DOI: 10.21203/rs.3.rs-62268/v1.

## Materials and methods

Patients and data collection

The Ohud Hospital and the King Fahd Hospital in the Madinah district of Saudi Arabia were used as research locations for this cross-sectional study. In the current investigation, a total of 100 people who had been diagnosed with COVID-19 and 90 healthy volunteers served as the control group, from April 28 to June 30, 2020. A sample size of 190 participants was estimated for this study.

Using the formula for a single cross-sectional, n = [z2 * p * q)/d2], the sample size was defined. Using the following parameters, the sample size was calculated: p = number of COVID-19 infected patients = 50%, Z = 95% confidence interval, d = error 5%, and 25% non-infected people (control).

The clinical and laboratory data that was utilized for the study were present within the patient records. All of the cases were looked at using the inclusion and exclusion criteria; the patients with confirmed instances of COVID-19 illness were used as the inclusion criterion. Patients who had a history of major renal, liver, heart, or neurological diseases, as well as those who had a history of endocrine diseases such as hyperthyroidism, hypothyroidism, adrenal disease, and pituitary disease, were not allowed to participate in the study.

The control group was healthy people who visited the hospital for routine investigation and their recording files revealed no serious renal, hepatic, cardiac, endocrine, or neurological disease in their medical history, and their ages were comparable to those of the patients in the study. All participants provided oral and written informed consent for participation in the present study was authorized by the Ethical Committee at the College of Applied Medical Sciences, Taibah University and General Administration for Research and Studies of the Saudi Arabia Ministry of Health (IRB 100-2021). According to the 2020 Saudi Health Ministry criteria, patients with proven COVID-19 infection were classified as having a high (severe), moderate, mild, or no risk of developing symptoms (asymptotic).

Blood Analysis

The liver function tests typically included alanine transaminase (ALT), aspartate transaminase (AST), and serum bilirubin. The renal function tests typically included creatinine, blood urea nitrogen (BUN), and electrolytes (Na=Sodium, K=Potassium, P=Phosphorus, Ca=Calcium, and Cl=Chloride).

Statistical analysis

GraphPad Prism, version 5.01 (GraphPad Software, San Diego, CA, USA) was utilized in order to do analysis on the data. The quantitative data were reported as percentages, means, standard deviations, and ranges. The data were assumed to follow a normal distribution. In order to compare the patients and the control group, a T-test was carried out. A one-way analysis of variance (Tukey's multiple comparisons test) was utilized in order to make a comparison between individuals with severe and mild or asymptotic COVID-19 infections. At the threshold of P ≤ 0.05 or 0.001, all of the differences were found to be statistically significant.

## Results

Patient characteristics

In the present study, 100 patients were diagnosed with COVID-19 with a mean age of 55.5±11.6 years (extremes: 30-80 years). The study subjects include 66 male and 34 female infected patients and 55 male and 35 female healthy participants with mean age of 54.5±10.2 (Table 1).

**Table 1:** Demographic characteristics of COVID-19 patients (n=100) and healthy people (n=90).

Variables	Number of COVID-19 patients (%)	Number of healthy people (%)
Age		
Mean±SD	55.5±11.6	54.5±10.2
30-39 years	11 (11%)	10 (11.1%)
40-49 years	26 (26%)	35 (38.9%)
50-59 years	22 (22%)	20 (22.3%)
60-69 years	26 (26%)	21 (23.3%)
70-79 years	6 (6%)	2 (2.2%)
80-89 years	9 (9%)	2 (2.2%)
Severity of classification		
Asymptotic	22 (22%)	Non
Mild	24 (24%)	
Moderate	27 (27%)	
Severe	27 (27%)	
Gender		
Male	66 (66%)	55 (61.1%)
Female	34 (34%)	35 (38.9%)
Smoking status		
Smoker	10 (10%)	5 (5.5%)
Non-smoker	90 (90%)	85 (94.5%)

Severity of COVID-19

According to the national protocol for patients with confirmed COVID-19 infection, all of the patients included in the study were classified into severe, moderate, mild, or asymptomatic based on the results from clinical features, chest radiography, and overall symptoms. Patients with mild symptoms and no abnormal laboratory findings were classified as asymptomatic. Severe cases were defined by respiratory rate >30/min, blood oxygen saturation <93%, oxygen partial pressure (PaO2)/fractional inspired oxygen (FiO2) ratio <300, and lung infiltrates >50% of the lung field within 24-48 hours. In the present study, out of 100 patients, 27 were severely infected, 22 were asymptomatic, and 51 were mild to moderately ill.

First Analysis

Compared to the control group, the patients with COVID-19 had high levels of liver enzymes (ALT and AST), bilirubin, and numerous elevated levels of creatinine, urea, Na, and Cl, while levels of K and albumin were lowered in the infected group compared to the control group (Table 2). Furthermore, a comparison between the infected and the control group (Table 2) revealed that the concentration of liver enzymes was higher significantly in COVID-19 patients when compared to the control group (P<0.001). Also, the levels of bilirubin were higher than the control group (P<0.001), whereas the levels of albumin showed significantly lower in the infected group when compared to the healthy control group (P=0.02). Additionally, there was a significantly higher level of creatinine, Na, Cl, and urea in COVID-19 patients compared to the control group (P<0.001). However, K and albumin levels were significantly lower in the COVID-19 patients compared to the control group (P<0.03 and 0.02 respectively, Table 2).

**Table 2:** Comparison between infected and non-infected patients on the basis of levels of liver and renal biomarkers Data represent the mean concentrations ± standard deviation for biochemical markers of the study population that were classified based on infected or not with COVID-19. *P < 0.05, **P < 0.01. AST=Aspartate Aminotransferase, ALT=Alanine Aminotransferase, BUN=Blood Urea Nitrogen, Na=Sodium, K=Potassium, P=Phosphorus, Ca=Calcium, Cl=Chloride

Parameters	COVID-19 patients (n=100)	Control (n=90)	P-Value
ALT (U/L)	65.22±50.91	12.8±8.6	0.001**
AST (U/L)	53.95±46.50	15.7±7.3	0.002**
Direct bilirubin (umol/L)	41.03±20.20	1.5±0.1	<0.001**
Bilirubin (umol/L)	50.89±32.62	3.4±1.5	<0.001**
Albumin (g/L)	29±9.35	40.7±31.9	0.02*
Creatinine (umol/L)	185.38±33.99	78.45±50.99	<0.001**
Urea (mmol/L)	19.59±14.36	3.27±1.30	0.002**
Uric acid (mmol/L)	0.30±0.31	0.27±0.12	>0.05
BUN (mol/L)	8.85±5.10	3.60±1.99	>0.05
Na (mmol/L)	148.03±15.81	138.17±78.99	0.04*
K (mmol/L)	1.25±0.92	3.99±2.92	0.03*
P (mmol/L)	1.08±0.69	0.84±0.30	>0.05
Ca (mmol/L)	2.21±0.47	2.30±1.99	>0.05
Cl (mmol/L)	111.95±11.67	105.30±9.99	0.02*

Second Analysis

A comparison of the liver biomarker results between the male and female COVID-19 patients using the T-test revealed a significant difference in the means between the two gender groups of COVID-19 patients. A statistically significant difference in ALT, AST and bilirubin levels (P<0.001) was found but no significant difference in albumin levels (P>0.05) was seen (Table 3). The comparison of the renal biomarkers of COVID-19 patients between males and females using T-test analysis showed a significant difference in the levels of creatinine, uric acid, BUN, K, and Cl between males and females (P<0.05) (Table 3).

**Table 3:** Comparison of levels of liver and renal biomarkers between male and female COVID-19 patients. Data were analyzed by T-test to compare between male and female patients with COVID-19 and a statistical significance difference was considered as P<0.05*. AST=Aspartate Aminotransferase, ALT=Alanine Aminotransferase, BUN=Blood Urea Nitrogen, Na=Sodium, K=Potassium, P=Phosphorus, Ca=Calcium, Cl=Chloride

Parameters	Female (n=34)	Male (n=66)	P-Value
ALT	22.38±11.07	30.8±12.6	0.05*
AST	20.3±11.27	55.7±13.98	0.04*
Direct bilirubin	38.33±15.83	60±49.1	0.01*
Bilirubin	15.64±7.80	87.1±22.5	0.03*
Albumin	9.4±9.11	8.7±5.9	0.09
Creatinine (umol/L)	172.95±17.63	102.67±20.98	0.04*
Urea (mmol/L)	17.53±12.58	10.11±6.16	0.08
Uric acid (mmol/L)	0.50±0.37	5.95±4.63	0.01*
BUN (mol/L)	6.36±5.29	68.05±8.63	0.02*
Na (mmol/L)	140.35±13.99	144.77±8.77	0.06
K (mmol/L)	3.83±1.1	1.77±0.77	0.05*
P (mmol/L)	1.17±0.92	0.50±0.46	0.07
Ca (mmol/L)	2.12±0.57	1.00±0.22	0.07
Cl (mmol/L)	112.13±10.57	108.12±11.57	0.04*

Third Analysis

Results of one-way ANOVA tests for differences in the levels of biomarkers between severe cases and mild or asymptomatic cases of COVID-19 are presented in Table 4. Additionally, we found that 7% of severely ill COVID-19 patients had proteinuria. There were significant differences in the levels of ALT, AST, albumin, creatinine, Na and Cl between severe cases and another two categories (asymptomatic and moderate) (P<0.001). Furthermore, there was a significant difference in the level of bilirubin between the severe and asymptotic cases, but none in mild cases (Table 4). Also, 13 (48.2%) out of 27 severe cases had renal damage and 12 (44.4%) had liver damage whereas two (7.4%) had both based on their blood biomarkers analysis (data not shown).

**Table 4:** A one-way between subject’s ANOVA was conducted to compare severe cases with asymptotic and mild cases in the levels of renal and liver markers. Mean Diff=Mean Difference, CI=confidence interval, *P<0.05, **P<0.01. AST=Aspartate Aminotransferase, ALT=Alanine Aminotransferase, BUN=Blood Urea Nitrogen, Na=Sodium, K=Potassium, P=Phosphorus, Ca=Calcium, Cl=Chloride.

Biomarkers	Asymptotic cases (n=22) Vs. severe cases (n=27)			Mild to moderate cases (n=51) Vs. severe cases (n=27)		
	Mean Diff.	95.00% CI of diff.	P	Mean Diff.	95.00% CI of diff.	P
ALT (U/L)	88.93	65.20 to 112.7	<0.001**	81.83	62.17 to 101.5	<0.001**
AST (U/L)	47.36	18.34 to 76.38	0.0005**	43.21	19.16 to 67.26	0.001**
Direct bilirubin (umol/L)	15.4	-12.36 to 43.16	0.3874	-5.433	-28.44 to 17.57	0.8405
Bilirubin (umol/L)	23.06	22.58 to 46.70	0.022*	-9.587	-38.29 to 19.11	0.707
Albumin (g/L)	17.36	2.34 to 20.68	0.021*	18.21	1.16 to 20.26	0.023*
Creatinine (umol/L)	30.62	16.2 to 44.2	<0.001**	24.1	12.6 to 35.7	<0.001**
Urea (mmol/L)	11.42	1.909 to 20.92	0.014*	6.977	-0.9008 to 14.85	0.093
BUN (mol/L)	1.629	-2.163 to 5.420	0.5646	-0.07495	-3.217 to 3.067	0.998
Na (mmol/L)	36.589	12.084 to 35.26	0.017*	26.388	5.7997 to 28.58	0.012*
K (mmol/L)	0.5402	-0.08487 to 1.165	0.1043	0.2274	-0.2906 to 0.7454	0.5504
Cl (mmol/l)	32.79	3.118 to 9.711	0.042*	20.179	1.722 to 8.080	0.0027**

## Discussion

In late December 2019, the Wuhan City of China experienced an unprecedented outbreak of a cluster of pneumonia cases having mysterious aetiology [6-8]. Since then, it has rapidly spread across China and to many other countries across the globe. Recent research on COVID-19 mainly emphasized on lungs as the main organ affected by the disease, while less data is reported regarding the involvement of other organs like the liver and kidneys. The involvement of multiple organs including the liver, gastrointestinal tract, and kidney has been previously reported in patients with COVID-19 [9].

Our study is perhaps unique in reporting the impact of the severity of COVID-19 disease on the liver and renal functioning of COVID-19 patients with no prior underlying liver or renal impairments. Recent studies have described distinctive levels of elevated liver enzymes in COVID-19 cases, mostly exhibiting higher ALT and AST levels accompanied by mildly elevated total bilirubin (TB) levels [10,11]. We have found similar kinds of results, wherein, the patients with elevated liver enzymes accompanied by moderate to highly elevated direct bilirubin depicted severe illness compared to those who had normal to very mildly elevated results. A similar kind of result was also reported in a study done by Cai et al. [12], where high levels of AST were seen in 62% (eight of 13) of patients who were critically ill and shifted to the intensive care unit (ICU) compared to 25% (seven of 28) of patients who were mildly ill having normal to mildly raised levels of liver enzymes and hence corroborating with our current findings. Furthermore, some of the asymptomatic COVID-19 patients in this study also showed normal or mildly elevated liver enzymes, suggesting that liver damage is more predominant in severe cases compared to mild cases of COVID-19 as also reported in a similar study done by Zhang et al. [13]. However, the reason for the elevation of liver enzymes or liver injury still remains unclear as to whether it is caused by the virus itself or due to a severe inflammatory reaction produced in response to the infection [14]. In this connection, a recent study by Hamming et al. revealed that the novel coronavirus might directly infect the liver cells as the docking receptor of the virus, angiotensin‐converting enzyme 2 (ACE2), is released by the cells of both liver and bile-duct, where the virus replicates and consequently invades the cells of the upper respiratory tract and lung tissue and hence the patients develop the clinical symptoms [15]. Moreover, a recent pilot study has proposed that an enhanced expression of the ACE2 receptor is developed in cholangiocytes [16] signifying that the novel coronavirus may bind directly to the ACE2-positive cholangiocytes and impair normal liver functions. Additionally, cytokine storm and pneumonia-related hypoxia might add to the liver damage leading to complete or partial liver failure in severely ill COVID-19 patients.

In addition to liver damage, an increased incidence of acute renal injury in COVID-19 patients has also been previously reported [17]. COVID-19 has been shown to impair renal function in several ways, ranging from poor blood flow to the formation of tiny blood clots which can clog the kidneys and prevent urine formation. The current clinical study revealed that patients suffering from severe cases of COVID-19 are also showing signs of kidney damage, even though they had no history of kidney ailments prior to the infection with the novel coronavirus.

We found the patients who were categorized as severely ill had altered kidney function tests, characterized by elevated serum creatinine, urea, and blood urea nitrogen levels as also reported by Fan et al. [18]. The altered kidney function can be attributed to the variations in ACE2 receptor expression leading to kidney dysfunction. ACE2 is expressed on the brush border apical membrane of the proximal tubules of kidneys, as well as in podocytes at low levels [19]. Therefore, it is plausible that the virus could enter the podocytes first, infect them, and then bind to ACE2 to enter the kidney tubular cells, which leads to impaired renal function, as has been reported by Batlle et al. [20].

Furthermore, we also observed hypernatremia and hyperchloremia in our severely ill COVID-19 patients, which has not been reported previously. We attribute the elevation of Na and Cl to severe water loss due to pyrexia, use of diuretics, and high respiration rate. Additionally, there are fair chances of hypernatremia and hyperchloremia in COVID-19 patients due to abnormally low levels of oxygen in the blood, caused by pneumonia which is common in severe cases of this disease. The large influx of cytokines known as cytokine storm leads to severe inflammation, which can damage healthy tissues, including that of the kidneys.

The current study has certain limitations, which include a limited number of patients, and the patient duration in the hospital was not enough to predict the possibility of remission of liver and renal damage. We were not able to detect the presence of SARS-CoV-2 in urine samples and hence could not evaluate the correlations between urine virus and kidney complications. Therefore, we recommend repeated and meticulous observation of liver and renal functions in patients with COVID-19 that can help in achieving the optimum mode of treatment and reduce the number of deaths due to organ failure.

## Conclusions

In conclusion, a significant number of patients with COVID-19 maintained their normal liver and kidney functions all through the course of their disease, however, patients who were severely ill had an effect on their liver and kidney functions and therefore had elevated ALT, AST, creatinine, urea, sodium, chlorine, and total bilirubin levels. All the COVID-19 patients enrolled in this study did not have any prior liver or kidney abnormalities and hence, this effect could possibly be attributed to the severity of COVID-19 infection. Therefore, further studies on the effect of COVID-19 infection on liver and kidney functions would be helpful in determining the course of treatment to avoid the associated complications.
